# Solvent-Regulated Surface Passivation for Efficient and Stable Inverted Perovskite Solar Cells

**DOI:** 10.3390/nano16181189

**Published:** 2026-09-21

**Authors:** Jiawei Wang, Boyuan Li, Yu Jiang, Jiaqi Du, Yongqi Yin

**Affiliations:** 1 Key Laboratory for Photonic and Electronic Bandgap Materials, Ministry of Education, School of Physics and Electronic Engineering, Harbin Normal University, Harbin 150025, China; 2024300795@stu.hrbnu.edu.cn (J.W.); 2025300790@stu.hrbnu.edu.cn (B.L.); 2024300767@stu.hrbnu.edu.cn (Y.J.); 2024300755@stu.hrbnu.edu.cn (J.D.); 2 Department of Materials Process Engineering, Graduate School of Engineering, Nagoya University, Furo-cho, Chikusa-ku, Nagoya 464-8603, Japan

**Keywords:** inverted perovskite solar cells, phenethylammonium iodide, solvent engineering, surface passivation, stability

## Abstract

Phenethylammonium iodide (PEAI) is widely used to passivate undercoordinated ionic defects and regulate the near-surface structure of inverted perovskite solar cells; however, the solvent used for PEAI deposition can also interact with the underlying perovskite and therefore determine the treatment outcome. Here, ethanol (EtOH), isopropanol (IPA), and *n*-butanol (*n*-BuOH) were compared as aliphatic alcohol solvents for the PEAI post-treatment of inverted p–i–n solar cells based on a mixed-cation, mixed-halide Cs_0.05_(FA_0.95_MA_0.05_)_0.95_Pb(I_0.95_Br_0.05_)_3_ absorber. EtOH caused pronounced surface disturbance and substantially reduced device performance. IPA afforded the highest initial power conversion efficiency (PCE) of 21.92% but was accompanied by a stronger PbI_2_ diffraction signal. In contrast, *n*-BuOH provided a milder treatment, producing a comparable PCE of 21.83%, a weaker PbI_2_ signal, and the highest water contact angle. After dark storage in air at 25 °C and 25% relative humidity for 168 h, the unencapsulated *n*-BuOH-treated devices retained 82% of their initial PCE, compared with 77% for the Control. These results demonstrate that the PEAI processing solvent is an active component that governs the balance among defect passivation, surface reconstruction, initial efficiency, and short-term storage stability.

## 1. Introduction

Perovskite solar cells have emerged as promising next-generation photovoltaic technologies because of their high power conversion efficiencies (PCEs), solution processability, tunable optoelectronic properties, and compatibility with low-temperature fabrication [1,2,3,4,5]. Among the different device architectures, inverted p–i–n perovskite solar cells have attracted particular interest because of their low current–voltage hysteresis, favorable operational stability, and compatibility with tandem photovoltaics. Nevertheless, defects at the perovskite surface and the perovskite/electron-transport-layer interface remain important sources of non-radiative recombination and degradation [6,7,8,9,10]. Polycrystalline perovskite films can contain undercoordinated ions, vacancies, residual precursor species, and structural imperfections at their surfaces and grain boundaries. These defects act as carrier-trapping and recombination centers, increasing open-circuit-voltage losses, hindering charge extraction, and accelerating degradation. Surface passivation is therefore widely used to suppress interfacial recombination and improve device performance and stability [11,12,13].

Organic ammonium halides are widely used as surface passivators because they can interact with undercoordinated ionic sites and regulate the near-surface structure of perovskite films. Phenethylammonium iodide (PEAI) can passivate surface defects and, under suitable processing conditions, promote the formation of a low-dimensional perovskite surface phase. At the ionic level, PEAI can interact with undercoordinated surface sites, reduce the surface defect density, and suppress trap-assisted non-radiative recombination [14,15,16]. The bulky PEA^+^ cation may also promote the formation of a low-dimensional PEA-based perovskite surface phase, while the organic-rich surface layer can improve resistance to moisture. However, excessive or spatially inhomogeneous surface reconstruction may impede charge transport or produce a heterogeneous interface. Therefore, the processing solvent plays an important role in determining the balance between defect passivation and surface reconstruction. Recent studies have demonstrated that the processing solvent can influence both perovskite surface reconstruction and passivator deposition. IPA-induced reconstruction toward a PbI_2_-rich surface has been reported, highlighting the direct involvement of the solvent in surface treatment [17]. Mixed-solvent strategies have subsequently been developed to balance passivator dissolution, surface modification, and deposition quality, including an IPA/chlorobenzene/THTO ternary formulation for 4-MeO-PEAI, with applicability also demonstrated for PEAI, and cosolvent-assisted blade coating of PEAI layers [18,19]. More recently, *n*-BuOH/DMSO formulations have been investigated for PEAI passivation of wide-bandgap perovskites, while low-hygroscopic alcohol/alkane mixtures have enabled ambient deposition of PEAI layers [20,21]. More broadly, solvent engineering has also been shown to regulate perovskite conversion and film formation during scalable processing [22]. During spin coating, the solvent not only transports the passivating species but also directly contacts the underlying perovskite. Differences in solvent polarity and volatility can therefore affect passivator distribution and perovskite surface reconstruction. Solvent selection should thus be considered an important part of the passivation strategy.

Alcohols are commonly used to deposit organic ammonium passivators because they can dissolve ionic salts and are readily removed during spin coating. Ethanol (EtOH) and isopropanol (IPA) are short-chain alcohols, whereas *n*-butanol (*n*-BuOH) has a longer carbon chain, lower polarity, lower water miscibility, and a higher boiling point. These differences can alter passivator solubility, solvent–perovskite interactions, surface residence time, and the extent of near-surface reconstruction. A direct comparison under otherwise identical processing conditions is therefore useful for identifying a suitable solvent window for PEAI treatment.

In this work, EtOH, IPA, and *n*-BuOH were compared as solvents for PEAI treatment of inverted perovskite solar cells. The comparison focused on how solvent choice affects the perovskite surface and the resulting device performance and stability. EtOH treatment strongly deteriorated the film and device properties. IPA produced the highest initial efficiency and exhibited a stronger PbI_2_ diffraction signal than the PEAI–*n*-BuOH film. *n*-BuOH yielded a comparable efficiency while limiting PbI_2_ formation, providing the highest surface hydrophobicity, and retaining 82% of the initial PCE after 168 h of storage. These findings identify *n*-BuOH as a suitable processing solvent for PEAI treatment in the investigated device system, balancing initial photovoltaic performance with limited surface alteration.

## 2. Materials and Methods

### 2.1. Materials

FTO-coated glass substrates, formamidinium iodide (FAI), lead(II) iodide (PbI_2_), C_60_, and bathocuproine (BCP) were purchased from Advanced Election Technology Co., Ltd., Yingkou, China. MeO-2PACz, PEAI, cesium iodide (CsI), methylammonium bromide (MABr), lead(II) bromide (PbBr_2_), and lead(II) thiocyanate [Pb(SCN)_2_] were purchased from Xi’an Yuri Solar Co., Ltd., Xi’an, China. N,N-Dimethylformamide (DMF), dimethyl sulfoxide (DMSO), chlorobenzene (CB), and IPA were purchased from Sigma-Aldrich, Shanghai, China. *n*-BuOH and EtOH were purchased from Aladdin, Shanghai, China. All materials were used as received unless otherwise stated.

### 2.2. Device Fabrication

FTO-coated glass substrates were sequentially cleaned by ultrasonication in laboratory detergent solution, deionized water, and ethanol, dried under nitrogen, and treated with UV–ozone for 15 min. MeO-2PACz (0.5 mg mL^−1^ in absolute ethanol) was spin-coated at 4000 rpm for 30 s and annealed at 100 °C for 10 min. The nominal precursor composition was Cs_0.05_(FA_0.95_MA_0.05_)_0.95_Pb(I_0.95_Br_0.05_)_3_, with Pb(SCN)_2_ added separately as an additive. The perovskite film was prepared by dissolving Pb(SCN)_2_ (1.9 mg), CsI (15.6 mg), MABr (6.4 mg), PbBr_2_ (22.5 mg), FAI (186.2 mg), and PbI_2_ (524.8 mg) in DMF/DMSO (0.75/0.25 mL), followed by stirring at 50 °C for 2 h and filtration through a 0.45 μm PTFE filter. The solution was spin-coated at 1000 rpm for 10 s and then at 5000 rpm for 40 s; 300 μL of CB was dispensed 28 s after the start of the second step. The films were annealed at 100 °C for 15 min. PEAI was dissolved in EtOH, IPA, or *n*-BuOH at a concentration of 0.50 mg mL^−1^ (~2.0 mM). The solutions were left to stand at room temperature for 1 h in a nitrogen-filled glovebox before spin coating. The same nominal PEAI concentration was used for all three solvents. All solutions were clear, with no visible precipitation before use. The solutions were then spin-coated on the perovskite layer at 4000 rpm for 30 s and annealed at 100 °C for 5 min [23]. C_60_ (60 nm), BCP (6 nm), and Ag (100 nm) were sequentially thermally evaporated. The active device area was 0.06 cm^2^. Solvent-only control samples were prepared by spin-coating neat EtOH, IPA, or *n*-BuOH onto the perovskite films at 4000 rpm for 30 s, followed by annealing at 100 °C for 5 min, without adding PEAI. The subsequent device fabrication steps were identical to those used for the PEAI-treated devices.

### 2.3. Characterization and Device Measurements

Surface morphologies were examined using field-emission SEM (ZEISS Gemini 560, ZEISS, Oberkochen, Germany). XRD patterns were recorded using a Rigaku SmartLab diffractometer (Rigaku, Tokyo, Japan) with Cu Kα radiation (λ = 1.54050 Å) over a 2*θ* range of 5–60° at 5° min^−1^. UV–vis absorption spectra were recorded using a JASCO V-770 spectrophotometer (JASCO, Tokyo, Japan) equipped with an integrating sphere. Steady-state PL spectra were collected using a HORIBA Fluoromax-4 fluorescence spectrometer (HORIBA, Kyoto, Japan) with an excitation wavelength of 460 nm. *J*–*V* characteristics were measured under simulated AM 1.5 G illumination (100 mW cm^−2^) using a 7-SS0503A solar simulator (Beijing SOFN Instruments Co., Ltd., Beijing, China) and a Keithley 2400 source meter (Keithley Instruments, Solon, OH, USA). The voltage was swept from −0.1 to 1.2 V with a 20 mV step and a 100 ms delay. *J*–*V* measurements were performed using a shadow mask with an aperture area of 0.08 cm^2^. Current densities were normalized to the 0.06 cm^2^ overlap area between the aperture and the Ag electrode. The light intensity was calibrated using a certified silicon reference cell. EQE spectra were recorded using a 7-SCSpec system (Beijing SOFN Instruments Co., Ltd., Beijing, China). Light-intensity-dependent *V*_oc_ measurements were performed over an illumination intensity range of 10–100 mW cm^−2^. The illumination intensity was varied using optical filters. The ideality factor (*n*) was extracted from the slope of *V*_oc_ versus ln(*I*) according to d*V*_oc_/dln(*I*) = *nk_B_T*/*q*, where *I* is the illumination intensity, *k_B_* is the Boltzmann constant, *T* is the measurement temperature, and *q* is the elementary charge. Static water contact angles were measured using an SZ-CAMC13 instrument (Shanghai Xuanzhun Instrument Co., Ltd., Shanghai, China). For storage-stability measurements, the unencapsulated devices were stored in air in the dark at 25 °C and 25% RH, and their photovoltaic performance was measured periodically over 168 h.

## 3. Results and Discussion

The mixed-cation, mixed-halide perovskite Cs_0.05_(FA_0.95_MA_0.05_)_0.95_Pb(I_0.95_Br_0.05_)_3_ films were deposited on FTO/MeO-2PACz substrates using an antisolvent-assisted spin-coating method. After complete crystallization, the films were post-treated with PEAI solutions of the same concentration prepared in EtOH, IPA, or *n*-BuOH. The untreated film is denoted as Control, whereas the corresponding PEAI-treated films are denoted as PEAI–EtOH, PEAI–IPA, and PEAI–*n*-BuOH, respectively. Detailed film preparation and post-treatment procedures are provided in Section 2.

**Figure 1:**
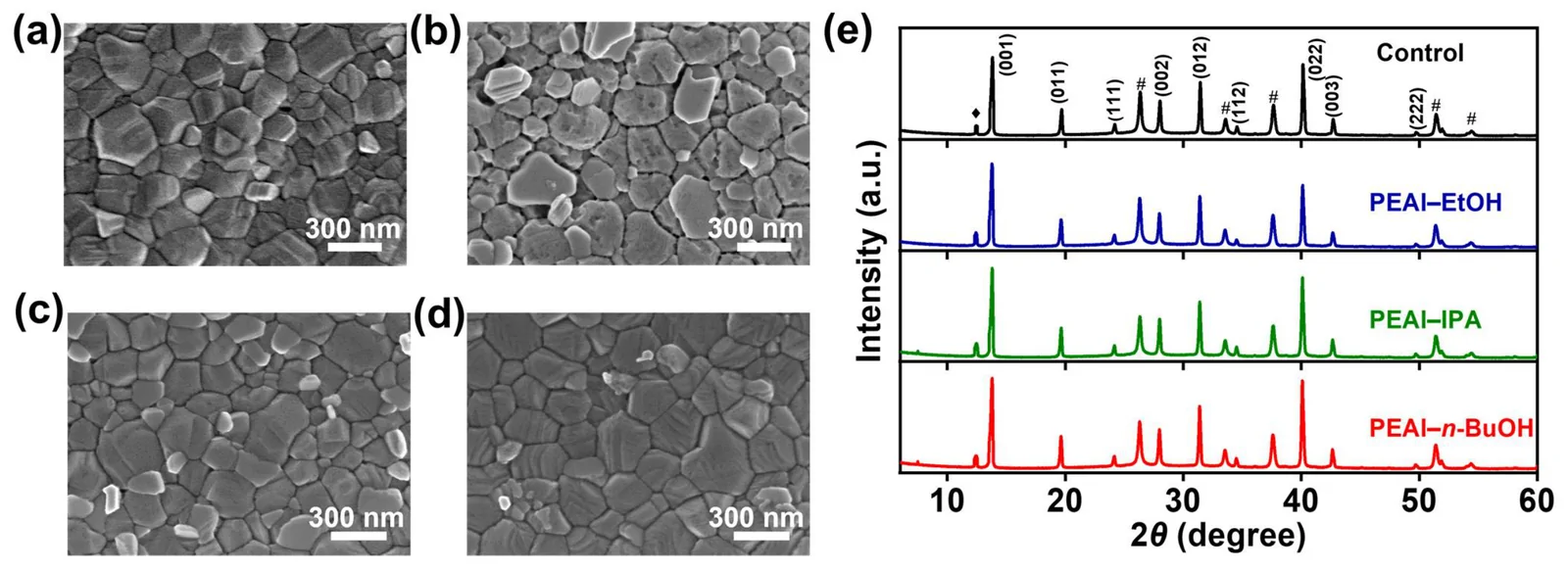
Top-view SEM images of (**a**) Control, (**b**) PEAI–EtOH, (**c**) PEAI–IPA, and (**d**) PEAI–*n*-BuOH perovskite films; (**e**) XRD patterns of the corresponding films. The symbols #, and ◆ denote reflections from the FTO substrate and PbI_2_, respectively.

The surface morphologies of the Control and PEAI-treated films were examined by top-view scanning electron microscopy (SEM), as shown in Figure 1a–d. The Control film exhibits a compact polycrystalline morphology with distinguishable grain boundaries and no obvious large-area pinholes. Clear solvent-dependent differences appear after PEAI treatment. The PEAI–EtOH film displays irregular grain contours and locally disturbed regions, indicating that the EtOH-based treatment alters the perovskite surface more strongly than the other treatments. In contrast, the PEAI–IPA and PEAI–*n*-BuOH films retain compact and continuous morphologies. Bright surface features are more evident in PEAI–IPA and may be associated with residual PbI_2_, based on the XRD results and previous reports [24]. PEAI–*n*-BuOH exhibits a more uniform surface with fewer bright features, suggesting that *n*-BuOH provides a milder environment for PEAI deposition. The grain-size distributions derived from the SEM images are shown in Figure S1. The Control film exhibits a relatively broad distribution, mainly between 150 and 250 nm, whereas the PEAI-treated films show distributions concentrated around 200–250 nm. The modest shift may reflect near-surface reconstruction or partial coalescence during post-treatment [25]. Because these modest changes were estimated from top-view SEM images, they should be regarded as changes in the apparent surface grain morphology rather than evidence of bulk grain growth.

The crystalline structures were examined by X-ray diffraction (XRD), as shown in Figure 1e. All samples retain the characteristic reflections of the three-dimensional perovskite phase. The reflections at approximately 13.9°, 19.8°, 24.3°, 28.1°, 31.4°, 34.6°, 40.1°, 42.7°, and 49.74° are assigned to the (001), (011), (111), (002), (012), (112), (022), (003), and (222) planes of the three-dimensional perovskite phase, respectively [26]. The absence of an obvious shift in the main reflections indicates that the bulk perovskite lattice is largely preserved after treatment. The PEAI–IPA film exhibits a distinctly stronger PbI_2_-related reflection at approximately 12.4°. Although a small amount of residual PbI_2_ might be beneficial to device performance through favorable interfacial energetics, excess or poorly distributed PbI_2_ can produce a heterogeneous interface and impair long-term stability [27]. The XRD result indicates that IPA-mediated PEAI treatment promotes the formation or exposure of PbI_2_-rich regions under the present processing conditions. PEAI–*n*-BuOH shows a substantially weaker PbI_2_ reflection, suggesting that *n*-BuOH better preserves the original perovskite phase while allowing surface passivation [28]. Because the reflections are weak, the phase assignment remains tentative. Overall, the SEM and XRD results show that EtOH causes the strongest surface disturbance, IPA combines PEAI treatment with enhanced PbI_2_ formation, and *n*-BuOH provides a more favorable balance between surface modification and preservation of the three-dimensional perovskite phase. Water contact-angle measurements were performed to evaluate the surface wettability of the perovskite films (Figure S2). The contact angle increases progressively from 60.0° for the Control to 83.0°, 89.2°, and 97.1° for PEAI–EtOH, PEAI–IPA, and PEAI–*n*-BuOH, respectively. The highest contact angle of PEAI–*n*-BuOH indicates reduced surface wettability, which may contribute to moisture resistance. These features indicate that the three solvents produce different extents or pathways of PEAI-induced surface reconstruction.

**Figure 2:**
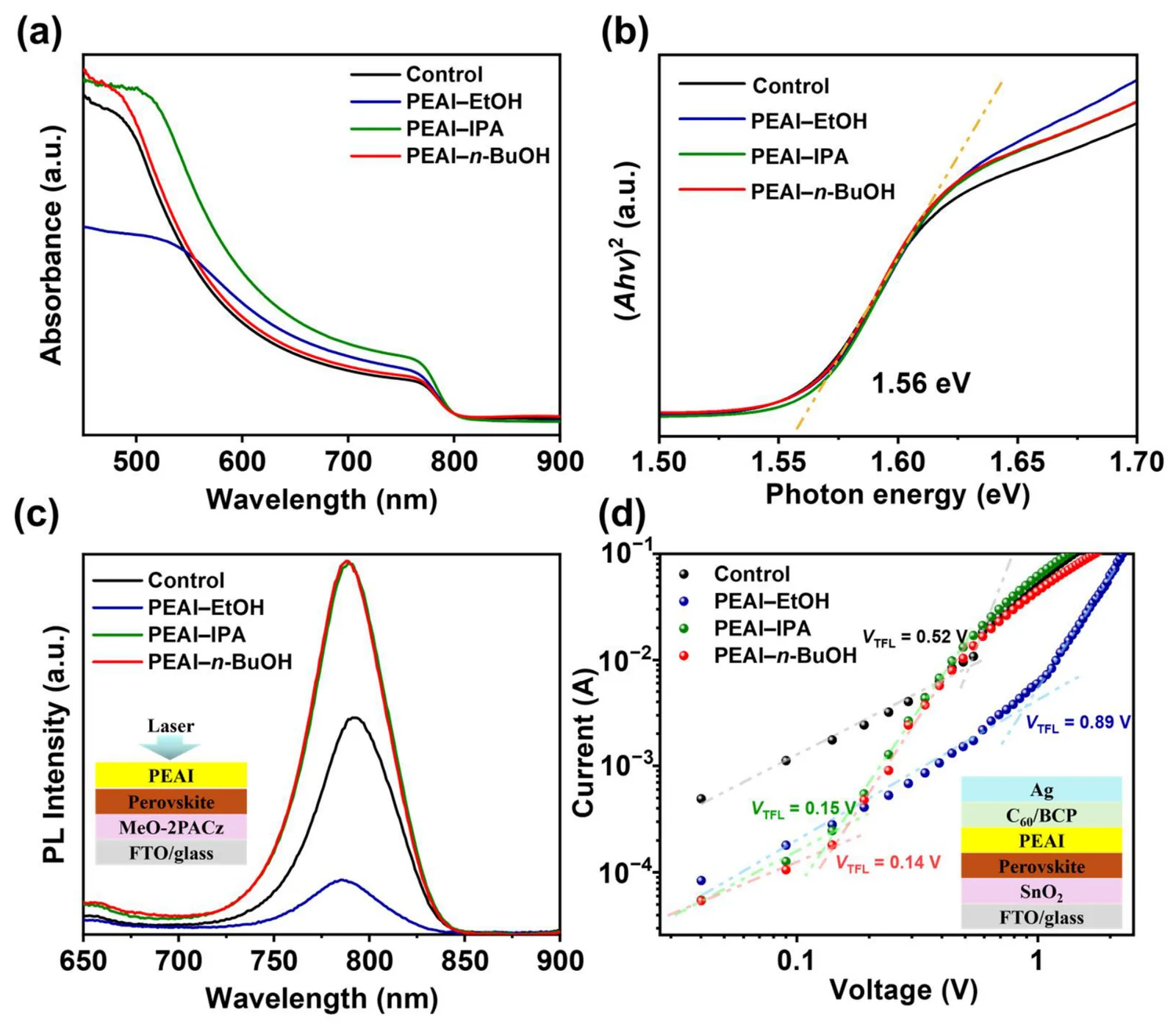
Optical properties and charge-trapping characteristics of the control and PEAI-treated samples. (**a**) UV–vis absorption spectra and (**b**) corresponding Tauc plots. (**c**) Steady-state PL spectra under 460 nm excitation; the inset shows the sample structure. (**d**) SCLC characteristics of electron-only devices; the inset shows the device architecture. The dashed lines indicatete linear fits used to extract the trap-filled-limit voltage (*V*_TFL_).

The optical absorption properties of the perovskite films were examined by ultraviolet–visible (UV–vis) spectroscopy. As shown in Figure 2a, all films exhibit a similar absorption edge near 790 nm without a discernible spectral shift, indicating that the different PEAI treatments do not substantially alter the optical bandgap of the perovskite absorber. Consistently, Tauc plots constructed for a direct allowed transition yield optical bandgaps of approximately 1.56 eV for all samples (Figure 2b). Although the PEAI–IPA film shows a higher apparent absorbance over much of the visible region, the PEAI–EtOH film exhibits a noticeably altered spectral profile and reduced absorption at shorter wavelengths, whereas the PEAI–*n*-BuOH film remains relatively close to the Control. Overall, these results demonstrate that the processing solvent influences the apparent absorbance of the films but has no measurable effect on their optical bandgap.

The steady-state photoluminescence (PL) spectra were further examined to evaluate the recombination behavior of the perovskite films (Figure 2c). The Control film exhibits an emission maximum at 793 nm, whereas the PEAI–EtOH, PEAI–IPA, and PEAI–*n*-BuOH films show emission maxima at 785, 789, and 788 nm, respectively. These modest blue shifts may be associated with changes in band-tail emission or near-surface composition induced by PEAI-mediated surface reconstruction [16,29]. The markedly quenched PL emission of PEAI–EtOH indicates increased non-radiative recombination, consistent with the pronounced surface disturbance observed by SEM. In contrast, PEAI–IPA and PEAI–*n*-BuOH exhibit substantially enhanced PL intensities, suggesting more effective suppression of non-radiative recombination following PEAI treatment. Notably, PEAI–*n*-BuOH achieves a PL intensity comparable to that of PEAI–IPA while exhibiting a weaker PbI_2_ diffraction signal. These results suggest that *n*-BuOH enables effective PEAI-mediated passivation without promoting the same extent of PbI_2_-related phase evolution observed for the IPA-treated film [30]. To further evaluate charge trapping, SCLC measurements were performed on electron-only devices with the structure FTO/SnO_2_/perovskite/PEAI/C_60_/BCP/Ag, with the PEAI treatment omitted for the control (Figure 2d). The trap density was estimated using the trap-filled-limit relation, $N_{t}=2\varepsilon_{r}\varepsilon_{0}V_{TFL}/(qL^{2})$. *ε*_0_ is the vacuum permittivity, *ε*_r_ is the relative dielectric constant, *q* is the elementary charge, and *L* is the perovskite thickness. A relative dielectric constant of 26 was adopted based on a previous study of an FA-rich mixed-halide perovskite [31], and a thickness of approximately 450 nm was used for all samples. The *V*_TFL_ values were 0.52, 0.89, 0.15, and 0.14 V for the control, PEAI–EtOH, PEAI–IPA, and PEAI–*n*-BuOH devices, respectively, corresponding to estimated trap densities of 7.38 × 10^15^, 1.26 × 10^16^, 2.13 × 10^15^, and 1.99 × 10^15^ cm^−3^. The lower estimated trap densities of the IPA- and *n*-BuOH-treated samples are consistent with their enhanced PL emission and support effective defect passivation.

**Figure 3:**
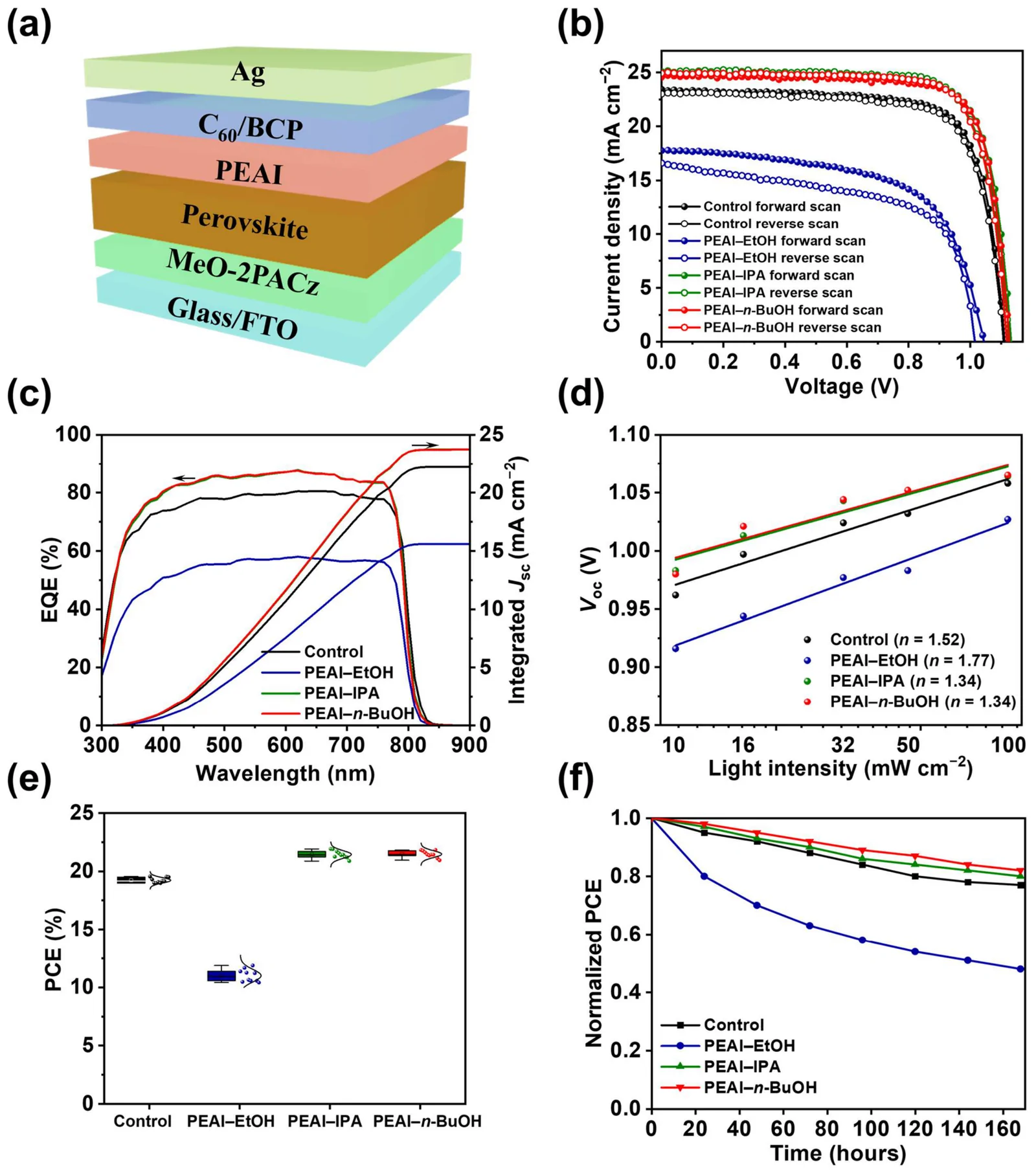
(**a**) Device architecture of FTO/MeO-2PACz/perovskite/PEAI/C_60_/BCP/Ag. (**b**) Forward and reverse *J*–*V* curves. (**c**) EQE spectra and corresponding integrated current densities. (**d**) Light-intensity-dependent *V*_oc_ and linear fits. (**e**) PCE distributions for 10 devices in each group. (**f**) Normalized PCE evolution of the unencapsulated Control, PEAI–EtOH, PEAI–IPA, and PEAI–*n*-BuOH devices stored in air in the dark at 25 °C and 25% RH for 168 h.

To evaluate the photovoltaic effects of the solvent-dependent PEAI treatments, inverted p–i–n PSCs were fabricated with the architecture FTO/MeO-2PACz/perovskite/PEAI/C_60_/BCP/Ag, with the PEAI layer omitted in the Control device. The device architecture is illustrated in Figure 3a, and detailed fabrication procedures are provided in Section 2. Cross-sectional SEM image of the devices are provided in Figure S3, showing perovskite layer thickness of approximately 450 nm. The forward and reverse current density–voltage (*J*–*V*) curves measured under simulated AM 1.5G illumination are shown in Figure 3b, and the corresponding photovoltaic parameters are summarized in Table S1.

The Control device delivers a PCE of 19.55%, with a *J*_sc_ of 23.43 mA cm^−2^, a *V*_oc_ of 1.11 V, and an FF of 75.14% in the forward scan. In comparison, the PEAI–EtOH device exhibits a substantially reduced PCE of 11.37%, with a *J*_sc_ of 17.75 mA cm^−2^, a *V*_oc_ of 1.04 V, and an FF of 61.32%. The simultaneous decreases across all photovoltaic parameters are consistent with the pronounced surface disturbance and PL quenching observed in the PEAI–EtOH film, indicating that the EtOH-based treatment yields an unfavorable perovskite surface and charge-transport interface. In contrast, the PEAI–IPA device achieves the highest PCE of 21.92%, with a *J*_sc_ of 24.79 mA cm^−2^, a *V*_oc_ of 1.13 V, and an FF of 78.11%. The PEAI–*n*-BuOH device delivers a comparable PCE of 21.83%, with a *J*_sc_ of 24.56 mA cm^−2^, a *V*_oc_ of 1.13 V, and the highest FF of 78.94%. The reverse scans showed the same PCE ranking, with efficiencies of 19.22%, 10.17%, 21.74%, and 21.66% for the control, PEAI–EtOH, PEAI–IPA, and PEAI–*n*-BuOH devices, respectively. The IPA- and *n*-BuOH-treated devices exhibited less pronounced *J*–*V* hysteresis than the EtOH-treated device under the measurement conditions used. Solvent-only controls yielded PCEs of 10.03%, 19.50%, and 19.52% for EtOH, IPA, and *n*-BuOH, respectively, compared with 19.55% for the untreated control (Figure S4 and Table S2). EtOH exposure alone therefore caused a pronounced performance loss, whereas the similar efficiencies of the IPA-only, *n*-BuOH-only, and untreated devices indicate that the improvements obtained with PEAI–IPA and PEAI–*n*-BuOH cannot be attributed to solvent treatment alone.

The enhanced PL intensities of these films are consistent with reduced non-radiative recombination following PEAI treatment. The stronger PbI_2_ diffraction signal of PEAI–IPA may also contribute to its initial photovoltaic performance, as an appropriate amount of residual PbI_2_ has been reported to passivate undercoordinated defects and suppress interfacial non-radiative recombination [32]. However, the influence of PbI_2_ depends strongly on its amount and spatial distribution, and its individual contribution cannot be separated from PEAI passivation based on the present results. Notably, PEAI–*n*-BuOH achieves a PCE comparable to that of PEAI–IPA while exhibiting a substantially weaker PbI_2_ diffraction signal. Together with its strong PL emission, this result suggests that *n*-BuOH enables effective PEAI-mediated surface passivation while limiting excessive PbI_2_-related phase evolution.

The external quantum efficiency (EQE) spectra are shown in Figure 3c. The integrated *J*_sc_ values are 22.22, 15.60, 23.74, and 23.72 mA cm^−2^ for the Control, PEAI–EtOH, PEAI–IPA, and PEAI–*n*-BuOH devices, respectively. These values reproduce the trend observed in the *J*–*V* measurements. PEAI–EtOH exhibits a reduced spectral response over most of the visible region, consistent with its low *J*_sc_ and poor device performance. In contrast, PEAI–IPA and PEAI–*n*-BuOH show enhanced EQE responses relative to the Control, resulting in comparable integrated current densities. Light-intensity-dependent *V*_oc_ measurements were performed to examine the recombination behavior of the devices (Figure 3d). The ideality factors extracted from the slopes of *V*_oc_ versus the natural logarithm of light intensity were 1.52, 1.77, 1.34, and 1.34 for the control, PEAI–EtOH, PEAI–IPA, and PEAI–*n*-BuOH devices, respectively. The lower ideality factors of the IPA- and *n*-BuOH-treated devices are consistent with reduced trap-assisted recombination, in agreement with the PL and SCLC results. Photovoltaic statistics for 10 devices per group are shown in Figure 3e and Figure S5, with the mean values and standard deviations summarized in Table S3. The average PCEs were 19.22 ± 0.23%, 11.01 ± 0.54%, 21.44 ± 0.33%, and 21.47 ± 0.29% for the control, PEAI–EtOH, PEAI–IPA, and PEAI–*n*-BuOH devices, respectively. Both IPA- and *n*-BuOH-based treatments improved the average PCE relative to the control and yielded comparable device efficiencies.

The storage stability of the unencapsulated devices was evaluated in air in the dark at 25 °C and 25% relative humidity (RH). The evolution of normalized PCE over 168 h is shown in Figure 3f. After 168 h, the Control device retains 77% of its initial PCE, whereas PEAI–EtOH degrades more rapidly and retains approximately 48%. The rapid degradation of PEAI–EtOH is consistent with its pronounced surface disturbance and strong PL quenching. In contrast, PEAI–IPA and PEAI–*n*-BuOH retain 80% and 82% of their initial PCEs, respectively, showing higher retention than the Control and PEAI–EtOH devices. Although the difference between PEAI–IPA and PEAI–*n*-BuOH is relatively small, the slightly higher retention of PEAI–*n*-BuOH is consistent with its higher water contact angle and weaker PbI_2_ diffraction signal. These results suggest that the milder *n*-BuOH-mediated treatment provides a favorable balance between PEAI passivation, preservation of the perovskite surface, and short-term dark-storage stability under the tested conditions.

## 4. Conclusions

This work investigated the influence of EtOH, IPA, and *n*-BuOH on PEAI-mediated surface treatment of inverted perovskite solar cells. The EtOH-based treatment caused pronounced surface disturbance and PL quenching, resulting in a substantially reduced PCE of 11.37%. The IPA-based treatment produced the highest initial PCE of 21.92% and exhibited a stronger PbI_2_ diffraction signal than *n*-BuOH. In comparison, the *n*-BuOH-based treatment yielded a comparable PCE of 21.83%, strong PL emission, a weaker PbI_2_ signal, and the highest water contact angle of 97.1°. After storage in air in the dark at 25 °C and 25% RH for 168 h, the unencapsulated PEAI–*n*-BuOH device retained 82% of its initial PCE, compared with 80% for PEAI–IPA and 77% for the Control. These results demonstrate that the processing solvent plays an important role in determining the outcome of PEAI treatment and that *n*-BuOH provides the most balanced combination of initial efficiency, surface preservation, and short-term storage stability under the tested conditions.

## Data Availability

The data presented in this study are available from the corresponding author upon reasonable request.

## Supplementary Materials

The following supporting information can be downloaded at: https://www.mdpi.com/article/10.3390/nano16181189/s1, Figure S1: Grain-size distributions derived from top-view SEM images of (a) Control, (b) PEAI–EtOH, (c) PEAI–IPA, and (d) PEAI–*n*-BuOH perovskite films; Figure S2: Water contact-angle images of (a) Control, (b) PEAI–EtOH, (c) PEAI–IPA, and (d) PEAI–*n*-BuOH perovskite films; Figure S3: Cross-sectional SEM image of control and PEAI–treated device with the architecture glass/FTO/MeO-2PACz/perovskite/PEAI/C60/BCP/Ag, (a) Control, (b) PEAI–EtOH, (c) PEAI–IPA, and (d) PEAI–*n*-BuOH; Figure S4: *J*–*V* curves of devices fabricated from untreated perovskite films and films treated with EtOH, IPA, or *n*-BuOH without PEAI; Figure S5: Statistical distributions of (a) *J*_sc_, (b) FF, and (c) *V*_oc_ for the control and PEAI-treated devices. Ten devices were measured for each condition; Table S1: Photovoltaic parameters of the champion devices measured in forward and reverse scans; Table S2: Photovoltaic parameters of devices fabricated from untreated perovskite films and films treated with EtOH, IPA, or *n*-BuOH without PEAI; Table S3: Photovoltaic parameters of the control and PEAI-treated devices, reported as mean ± standard de-viation for 10 devices per group.
