# Supplementary material for: Solvent-Regulated Surface Passivation for Efficient and Stable Inverted Perovskite Solar Cells
Source: Nanomaterials (Basel). 2026 Sep 21;16(18):1189. doi: 10.3390/nano16181189 (PMC13610465; doi:10.3390/nano16181189)
Supplement: Supplementary file 1 [file nanomaterials-16-01189-s001.zip › nanomaterials-4548363-supplementary.pdf]

Supplementary Materials

Solvent-Regulated Surface Passivation for Efficient and Stable Inverted Perovskite Solar Cells

Jiawei Wang [1,†], Boyuan Li [1,†], Yu Jiang [1], Jiaqi Du [1] and Yongqi Yin [1,2,*]

1 Key Laboratory for Photonic and Electronic Bandgap Materials, Ministry of Education, School of Physics and Electronic Engineering, Harbin Normal University, Harbin 150025, China; 2024300795@stu.hrbnu.edu.cn (J.W.); 2025300790@stu.hrbnu.edu.cn (B.L.); 2024300767@stu.hrbnu.edu.cn (Y.J.); 2024300755@stu.hrbnu.edu.cn (J.D.)

2 Department of Materials Process Engineering, Graduate School of Engineering, Nagoya University, Furo-cho, Chikusa-ku, Nagoya 464-8603, Japan

* Correspondence: yin.yongqi.i8@f.mail.nagoya-u.ac.jp

† These authors contributed equally to this work.

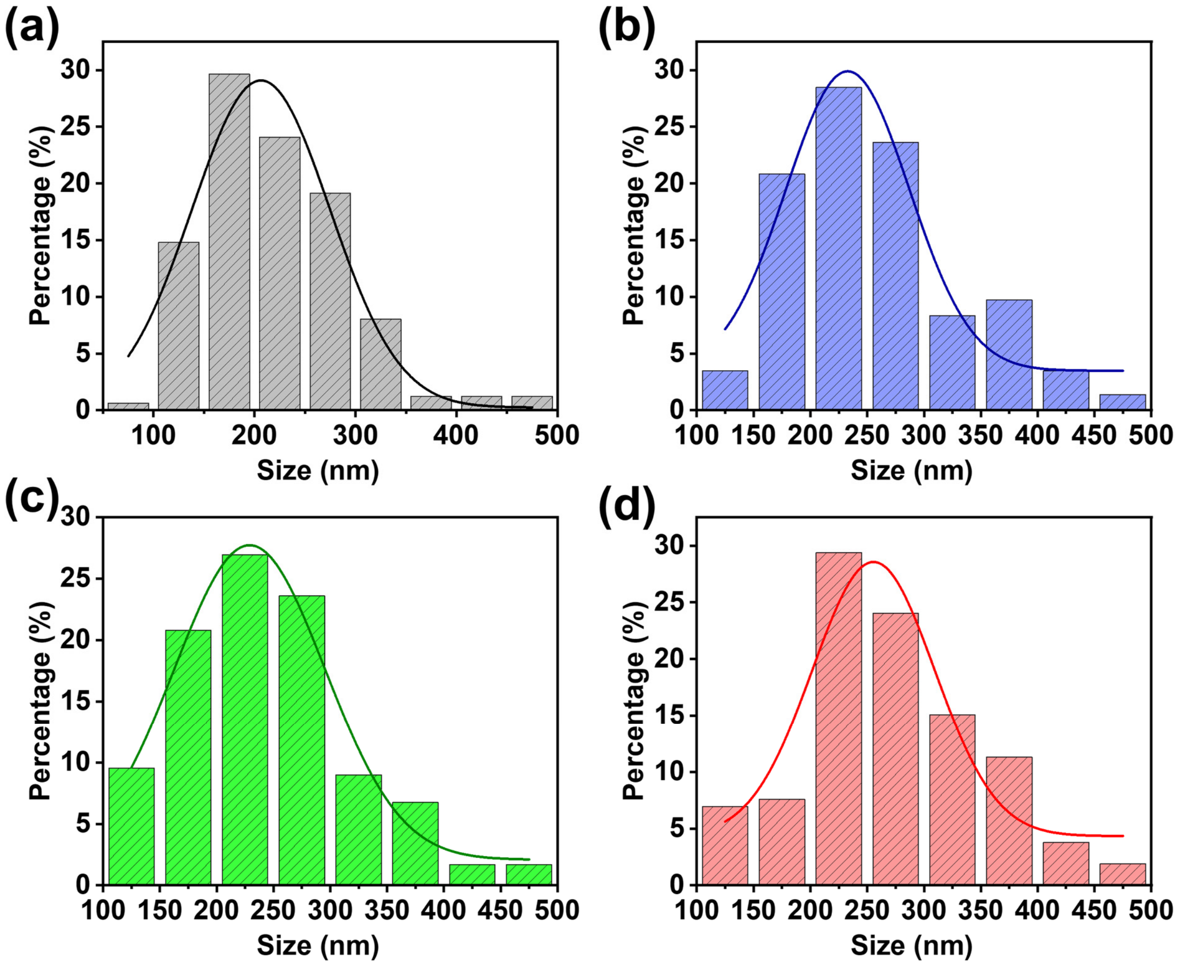

Figure S1. Grain-size distributions derived from top-view SEM images of (a) Control, (b) PEAI–EtOH, (c) PEAI–IPA, and (d) PEAI–*n*-BuOH perovskite films.

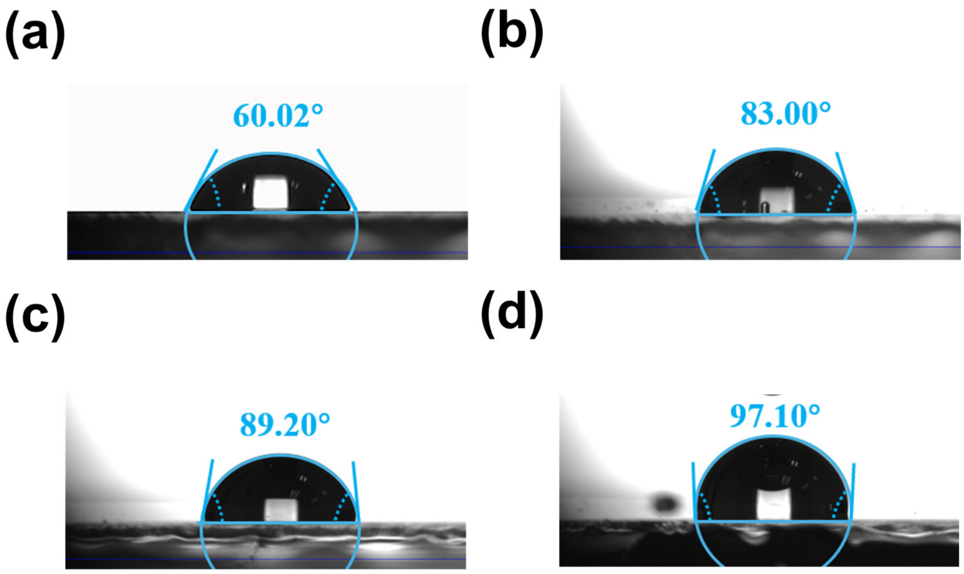

Figure S2. Water contact-angle images of (a) Control, (b) PEAI–EtOH, (c) PEAI–IPA, and (d) PEAI–*n*-BuOH perovskite films.

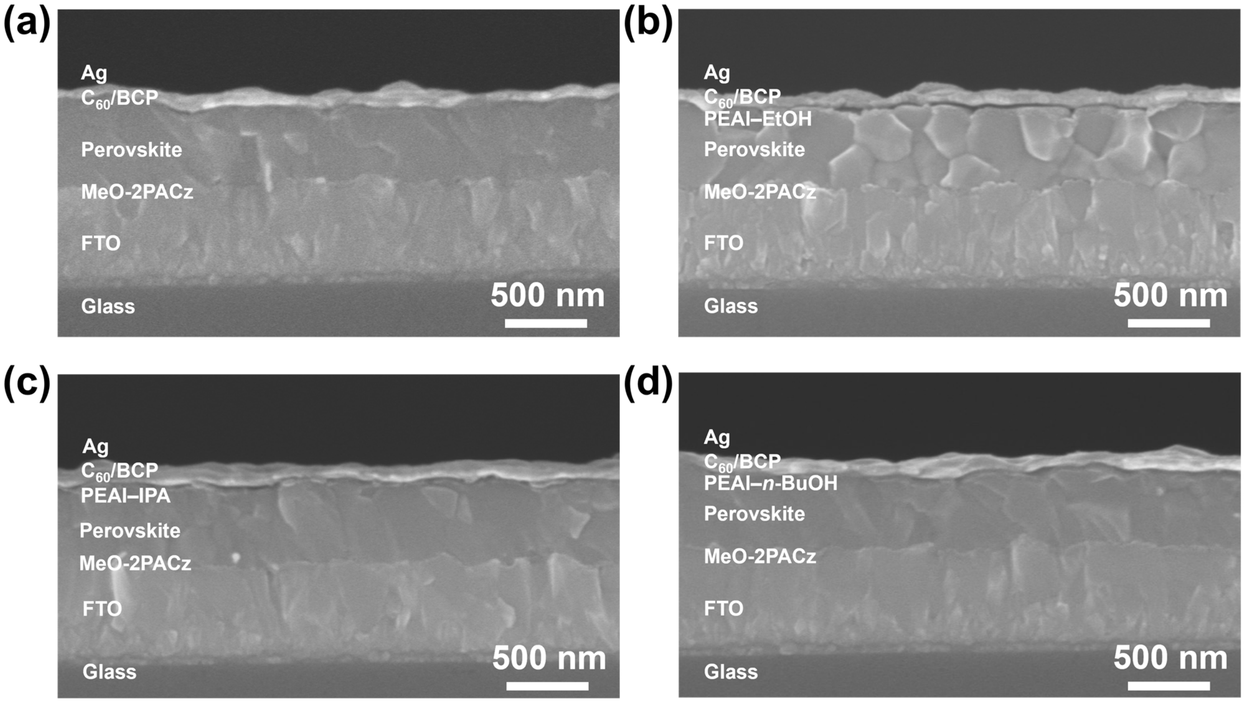

Figure S3. Cross-sectional SEM image of control and PEAI–treated device with the architecture glass/FTO/MeO-2PACz/perovskite/PEAI/C_{60}/BCP/Ag, (a) Control, (b)PEAI–EtOH, (c) PEAI–IPA, and (d) PEAI–*n*-BuOH.

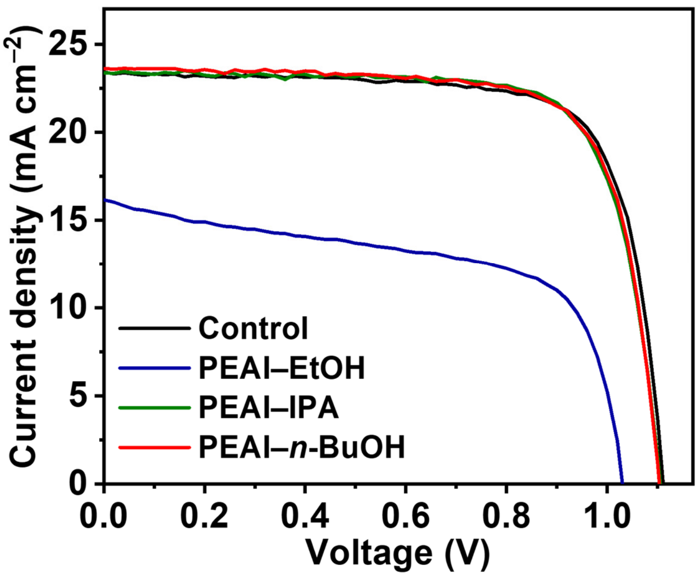

Figure S4. *J*–*V* curves of devices fabricated from untreated perovskite films and films treated with EtOH, IPA, or *n*-BuOH without PEAI.

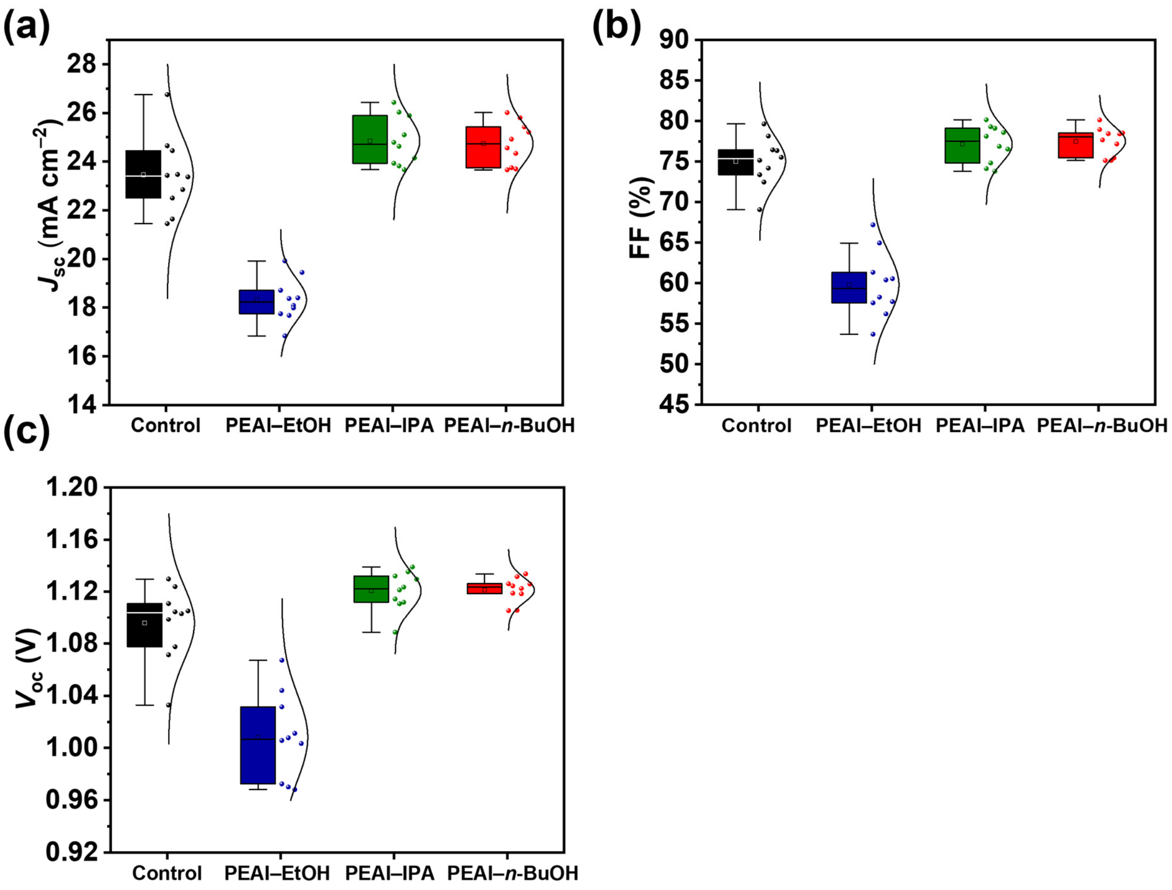

Figure S5. Statistical distributions of (a) J_{sc}, (b) FF, and (c) V_{oc} for the control and PEAI-treated devices. Ten devices were measured for each condition.

Table S1. Photovoltaic parameters of the champion devices measured in forward and reverse scans.

Devices	J_{sc} (mA cm^{-2})	V_{oc} (V)	FF (%)	PCE (%)
Control-forward scan	23.43	1.11	75.14	19.55
Control-reverse scan	23.01	1.11	75.40	19.22
PEAI–EtOH-forward scan	17.75	1.04	61.32	11.37
PEAI–EtOH-reverse scan	16.60	1.01	60.41	10.17
PEAI–IPA-forward scan	24.79	1.13	78.11	21.92
PEAI–IPA-reverse scan	25.05	1.12	77.16	21.74
PEAI–*n*-BuOH-forward scan	24.56	1.13	78.94	21.83
PEAI–*n*-BuOH-reverse scan	24.92	1.12	77.71	21.66

Table S2. Photovoltaic parameters of devices fabricated from untreated perovskite films and films treated with EtOH, IPA, or *n*-BuOH without PEAI.

Devices	J_{sc} (mA cm^{-2})	V_{oc} (V)	FF (%)	PCE (%)
Control	23.43	1.11	75.14	19.55
EtOH	16.15	1.03	60.3	10.03
IPA	23.37	1.11	75.51	19.5
n-BuOH	23.62	1.10	74.9	19.52

Table S3. Photovoltaic parameters of the control and PEAI-treated devices, reported as mean ± standard deviation for 10 devices per group.

Devices	J_{sc} (mA cm^{-2})	V_{oc} (V)	FF (%)	PCE (%)
Control	23.46±1.55	1.09±0.03	75.01±2.99	19.22±0.23
PEAI–EtOH	18.13±0.89	1.01±0.03	59.78±4.03	11.01±0.54
PEAI–IPA	24.84±0.99	1.12±0.01	77.12±2.27	21.44±0.33
PEAI–*n*-BuOH	24.74±0.87	1.12±0.01	77.49±1.75	21.47±0.29
